# Reversible Oxygenation of *α*-Amino Acid–Cobalt(II) Complexes

**DOI:** 10.1155/2016/3585781

**Published:** 2016-02-28

**Authors:** Xincun Zhang, Fan Yue, Hui Li, Yan Huang, Yi Zhang, Hongmei Wen, Jide Wang

**Affiliations:** ^1^Key Laboratory of Oil and Gas Fine Chemicals, Ministry of Education and Xinjiang Uyghur Autonomous Region, College of Chemistry and Chemical Engineering, Xinjiang University, Urumqi, Xinjiang 830046, China; ^2^College of Chemistry and Chemical Engineering, Central South University, Changsha, Hunan 410083, China

## Abstract

We systematically investigated the reversibility, time lapse, and oxygenation-deoxygenation properties of 15 natural *α*-amino acid–Co(II) complexes through UV-vis spectrophotometer, polarographic oxygen electrode, and DFT calculations, respectively, to explore the relationship between the coordinating structure and reversible oxygenation of *α*-amino acid–Co(II) complexes. Results revealed that the *α*-amino acid structure plays a key role in the reversible oxygenation properties of these complexes. The specific configuration of the *α*-amino acid group affects the e_g_^1^ electron of Co(II) transfer to the *π* ^⁎^ orbit of O_2_; this phenomenon also favors the reversible formation and dissociation of Co–O_2_ bond when O_2_ coordinates with Co(II) complexes. Therefore, the co-coordination of amino and carboxyl groups is a determinant of Co complexes to absorb O_2_ reversibly. The group adjacent to the *α*-amino acid unit evidently influences the dioxygen affinity and antioxidation ability of the complexes. The presence of amino (or imino) and hydroxy groups adjacent to the *α*-amino acid group increases the oxygenation-deoxygenation rate and the number of reversible cycles. Our findings demonstrate a new mechanism to develop reversible oxygenation complexes and to reveal the oxygenation of oxygen carriers.

## 1. Introduction

Oxygenated complexes should be investigated whether as a model compound of natural oxygen carriers or as an environmentally friendly catalyst [1–6]. The oxygenation, related mechanism, and configuration of oxygenated complexes as a model compound of natural oxygen carriers have been extensively explored [7–13]. Nam synthesized crystals of mononuclear oxygenated complexes and speculated their oxygenation mechanism by systematically investigating their aging process, that is, the process of activation of dioxygen and then the oxidation of the oxygenated complex [1, 14–17]. For example, metalloenzymes activate dioxygen to perform various biological reactions [8, 10, 18–20]. The activation of dioxygen at enzyme active sites occurs through several steps: (1) O_2_ binds to a reduced metal center; (2) superoxo and peroxo species are then generated; the O–O bond of metal hydroperoxo complexes is cleaved; and high-valent metal-oxo oxidants are formed [2, 3, 21–26]. Studies have demonstrated the mechanisms by which biological enzymes activate oxygen molecules. However, reversible oxygenation complexes, which can be used as a model compound of hemoglobin, have been rarely reported because available oxygenation complexes are unstable; as a result, researchers experience difficulty in characterizing the exact structures of products [27, 28]. Two major problems are encountered in studies involving the oxygenation of cobalt complexes in an aqueous solution: (1) complexes absorb dioxygen immediately when they are formed under ambient conditions and (2) aging phenomenon or the oxidation of the oxygenated complex occurs until the aging process is completed [29, 30]. Although the rates of the aging or oxidation of these cobalt complexes differ, oxygenated complexes exist in varying states. As such, oxygenated compounds should be characterized immediately after these compounds are formed in solutions rather than after they become separated from solutions. UV-vis spectrophotometer and polarographic oxygen electrode are used to monitor oxygenation and deoxygenation online through the continuous alternation of O_2_ and N_2_ atmospheres. A mass spectrometer is utilized to characterize complexes and to determine their structures [29–31].

Schiff base ligands and porphyrins are mostly confined in the reversible oxygenation of oxygenated complexes [11, 24]; however, the reversible oxygenation property of these complexes is poor in aqueous solutions and at room temperature. Burk et al. investigated histidine–Co(II) and demonstrated that oxygenation reversibility occurs in an aqueous solution at room temperature [32]. Considering this previous study, Martell and other researchers examined Co(II) complexes with amino acids and dipeptides, which can reversibly uptake oxygen [33, 34]. In our study, four groups of ligands with different N/O, N/N, and O/O difunctional groups [35], such as amino acids, amino alcohols, polyamines, and multicarboxylic acids, were selected. The oxygenation performances of their Co(II) complexes were comparatively analyzed. The following results were obtained: the O/O difunctional group of the Co(II) complexes does not possess oxygenation properties; the N/N difunctional group of the complexes can uptake oxygen but cannot undergo reversibility; the N/O-type difunctional group of the complexes is relatively different; *α*-amino acid–Co(II) complexes exhibit evident reversible oxygen performance; and *α*-amino alcohol–Co(II) complexes uptake oxygen weakly and show no reversibility. However, studies have yet to determine the factor causing *α*-amino acid–Co complexes to exhibit oxygenation reversibility.

Among Co complexes, *α*-amino acid–Co complexes display different oxygenation reversibilities. On the basis of this unusual finding, we supposed that the *α*-amino acid group in a ligand could be a key group associated with the reversible oxygenation of such complexes. Therefore, we synthesized a series of novel amino acid–Co(II) complexes and found that Co complexes can reversibly uptake dioxygen [28, 35, 36]. Indeed, the *α*-amino acid unit in a ligand is a key structural component related to the reversible oxygenation property of these complexes.

**Scheme 1:**
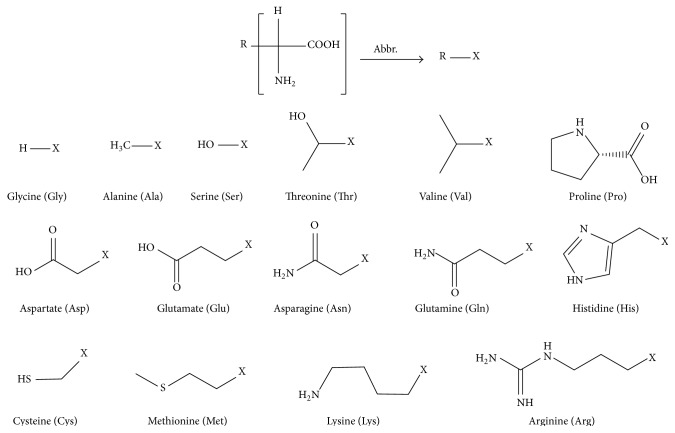
Structures of 15 natural amino acids.

To explore the factors and coordinating structure that determine the oxygenation reversibility of *α*-amino acid–Co complexes, we selected 15 natural water-soluble *α*-amino acids (Scheme 1) and performed a detailed investigation of their oxygenation properties in aqueous solutions at room temperature. Measurements were conducted at a specific pH range at which these complexes are in their major states. We also conducted a thorough comparative analysis to determine the relationship between the structures of ligands and the dioxygen affinity of Co complexes.

This study aimed to determine the basic structural unit responsible for the reversible dioxygen uptake. This study also aimed to identify the auxiliary functional groups that improve the reversibility of dioxygen uptake. We believe that our findings would remarkably contribute to elucidating and revealing the oxygenation mechanism of oxygen carriers.

## 2. Experimental

### 2.1. Materials

Amino acids and Co(CH_3_COO)_2_·4H_2_O were purchased from Shanghai Aladdin Reagents Co., Ltd. (China). Amino acids and Co aqueous solutions were prepared with distilled water. High-purity (99.99%) O_2_ and N_2_ were used. All of the chemicals were used without further purification.

The following instruments were used in the experiment: UV-vis spectrometer (UV-2450, Shimadzu, Japan), infrared spectrometer (VERTEX70-RAMAN II, Bruker Company, Germany; test conditions: dpi: 4.0 cm^−1^, number of scans: 100, and ATR with water as background), peristaltic pump (PS19-2, Pgeneral, China) (PP2), portable dissolved oxygen meter (HI 9146, Hanna Instruments, Italy), and pH Meter (PHS-3C, Shanghai Shengci Instrument Co., China).

### 2.2. UV-vis Spectrophotometry

Table 1 provides a list of the concentrations of the *α*-amino acids and Co(II) to prepare the corresponding complex solutions. The spectra were recorded at 25.0 ± 0.1°C by using a UV2450 spectrophotometer with a 1 cm cuvette within the spectral range of 200–600 nm or at the maximum absorption peak (*λ*_max_) of each complex at a certain pH.

### 2.3. Mass Spectrometry

Mass spectrometry was performed with Waters Quattro Premier XE mass spectrometer equipped with an electrospray ionization source (Micromass, Manchester, UK).

### 2.4. Construction of the Absorption (*A*)-pH Curves

For each amino acid, the Co(II) solution with a known concentration was mixed with the *α*-amino acid solution at a molar ratio of 1 : 3 or 1 : 2, depending on the *α*-amino acid species (Table 1). The *A* versus pH curve was constructed in accordance with a previously described method [28], and the suitable pH ranges to examine each complex were selected from the *A*-pH curves.

### 2.5. Determination of the Reversibility of Dioxygen Uptake and Release

The oxygenation and deoxygenation kinetics were determined using a PP2 flow injection apparatus [28]. The reversibility of the oxygenation and deoxygenation of the *α*-amino acid–Co(II) complexes was identified by recording the changes in the absorbance of O_2_ and N_2_ saturated solutions. The absorbance difference (Δ*A* = *A*_O_ − *A*_N_) between the absorbances in O_2_  (*A*_O_) and N_2_ atmospheres (*A*_N_) was considered to evaluate the ability of the complexes to uptake O_2_. The number of oxygenation-deoxygenation cycles (*C*) was obtained to estimate the endurance of each complex to antioxidation.

### 2.6. Oxygenometry

The concentration of the dissolved O_2_ in the solution corresponded to the evolution of the oxygenation of the complexes. The concentration of the dissolved O_2_ in the solution was measured in accordance with a previously described method [28].

### 2.7. DFT Calculation

Calculations were performed with the Gaussian 03W program package [37]. Full geometry optimization computations were conducted via a B3LYP method. In all of the calculations, a LANL2DZ basis set, along with the corresponding effective core potential, was used for Co metal atoms. The 6-31G(d) basis set was utilized for C, H, N, and O atoms.

## 3. Results and Discussion

The oxygenation and reversible performances of the 15 *α*-amino acid–Co complexes were investigated in this paper. The Ala–Co complex was used as an example to demonstrate the experimental processes.

### 3.1. Complex Formation

#### 3.1.1. UV-vis Spectra

Figure S1A (in Supplementary Material available online at http://dx.doi.org/10.1155/2016/3585781) shows the UV-vis spectra of Ala and Co(II) salt solution alone and their mixtures. The spectra of the mixture are distinctly different from those of Co or ligands alone; this result confirmed that the complexes were formed. Similar results were observed in the other amino acid–Co systems, as indicated by the UV-vis spectra. Figure S1 presents the UV-vis spectra of Ser–Co, His–Co, and Lys–Co.

#### 3.1.2. IR Spectroscopy

Figure S2 shows the IR spectra of L and Ala–Co(II), Ser–Co, His–Co, and Lys–Co. Clearly, the spectra of these complexes are significantly different from those of amino acids alone, which refers to the formation of the complexes.

#### 3.1.3. Mass Spectrometry Analysis

All amino acid–Co complexes were determined by MS, and the results exhibited the formation of the complexes. Figures S3 and S4 present the ESI mass spectrum of His–Co and Ala–Co.

### 3.2. Determination of the Suitable pH Condition for the Formation of Each *α*-Amino Acid–Co Complex

Co(II) complexes could be formed at different pH because of the differences in the coordinating abilities of amino acids to Co(II). *A*-pH curves of all the complexes were recorded by UV-vis spectrophotometry according to the part of experiment. The suitable pH for each complex was selected according to these *A*-pH curves.  Table 2 lists the suitable pH for the formation of oxygenated complexes and *λ*_max_.

Figure S5 presents the figure of *A*-pH curves for Ala–Co (curve 1), Ser–Co (curve 2), His–Co (curve 3), and Lys–Co (curve 4). The suitable pH for the formation of these complexes was concluded to be as follows: 9.3–1.2 (Ala–Co), 9.5–9.8 (Ser–Co), 7.8–10.3 (His–Co), and 10.3–10.8 (Lys–Co); their compositions were also determined via molar ratio method in corresponding pH, and their formulae are Co(Ala)_3_, Co(Ser)_2_, Co(His)_2_, and Co(Lys)_3_, respectively. Other amino acid–Co systems were tested in the same method.

### 3.3. Determination of Reversibility for the Uptake and Release of Dioxygen

**Figure 1:**
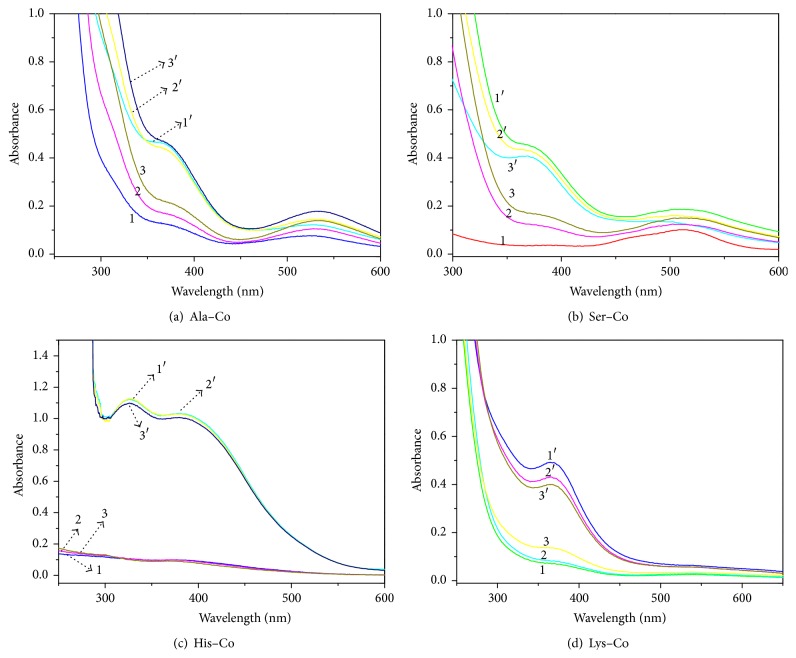
Reversible performance of L–Co was determined by UV-vis spectrophotometry (curves 1, 2, and 3 were tested in nitrogen, and curves 1′, 2′, and 3′ were tested in an oxygen atmosphere).

In the N_2_ atmosphere, when Ala and Co(II) salt solutions were mixed at pH 9.5, the Ala–Co complex solution showed a distinct spectrum with two main absorption peaks at 365 and 540 nm in an aqueous solution, thereby indicating the formation of the complex (Figure 1(a), curve 1). When dioxygen was added, the absorption intensity of the Ala–Co complex increased abruptly at 365 nm (Figure 1(a), curve 1′), and the color of the solution rapidly changed from light pink to orange-yellow, hence indicating that Ala–Co could be easily oxygenated in an aqueous solution at room temperature. This spectral change is caused by the charge transfer from oxygen to Co(II) (LMCT) [24]. When the atmosphere was changed from dinitrogen to dioxygen and subsequently back to dinitrogen (defined as one cycle), the spectrum changed regularly according to the change of the gas atmosphere (Figure 1(a), curves 2 and 3 for N_2_ and 2′ and 3′ for O_2_). These results confirmed that the oxygenation-deoxygenation reactions of Ala–Co are reversible. Other *α*-amino acid–Co complexes were tested in the same manner. The spectral changes of Ser–Co, His–Co, and Lys–Co displayed that the oxygenation of these three cobalt complexes was reversible (Figures 1(b), 1(c), and 1(d)). The spectra of other cobalt complexes dropped evidently after three cycles, but the spectra of His–Co remained the same after 15 cycles. These results showed that autoxidation occurred during oxygenation, and His–Co had an excellent reversible oxygenation ability.  Table 2 provides the results of reversibility for all the 15 amino acid complexes.

### 3.4. Oxygenometry Method

Evolution of the dissolved dioxygen concentration in airtight complex solutions was examined using a dissolved oxygen meter within the pH range of 3–11 and subsequently from 11 back to 3 at 25.0 ± 0.1°C. Figure S6 exhibits the diagrams of the dissolved dioxygen concentration as a function of pH (from 3 to 11 and back to 3) of the Ala–Co, Ser–Co, His–Co, and Lys–Co complexes. These concentration curves of the dissolved dioxygen in oxygenation coincided well with those of deoxygenation, thus indicating that the oxygenation of the complex is reversible.

The reversibility of oxygenation for other *α*-amino acid–Co complexes was also examined in the same procedure, and the results are listed in Table 2. The results obtained from oxygenometry also agreed well with those obtained from UV-vis spectrophotometry.

The 15 *α*-amino acid complexes have affinities to dioxygen, and 14 of them could reversibly bind dioxygen at suitable pH values; however, Cys–Co could only take oxygen but did not release it (Table 2).

### 3.5. Dynamics of Oxygenation-Deoxygenation of the *α*-Amino Acid–Co Complexes

**Figure 2:**
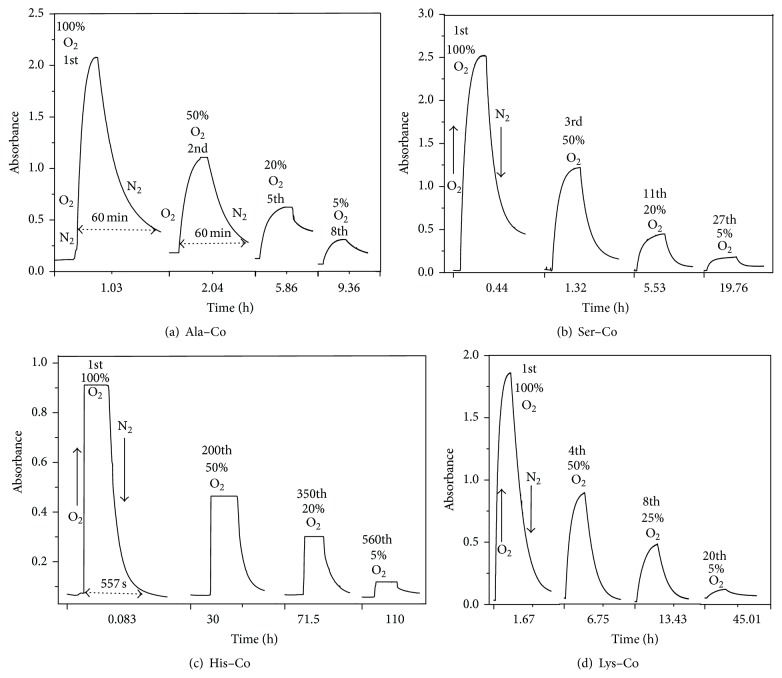
Absorbance changes at *λ*_max_ when N_2_ and O_2_ were alternately introduced.

After three oxygenation-deoxygenation cycles, the Ala–Co(II) complex still maintained reversible performance. The time-dependent cycle numbers of oxygenation-deoxygenation of Ala–Co(II) were determined to elucidate the complete oxygenation process and antiaging ability of the oxygenated complex. The time elapsing changes of absorbance were recorded as N_2_ and O_2_ were alternately bubbled into the system to observe the evolution of the oxygenation species. The difference of absorbance (Δ*A*) under N_2_ and O_2_ in one cycle was used to identify the oxygenation ability of the complex.  Figure 2 represents the oxygenation-deoxygenation kinetics of the Ala–Co(II) complex. The Ala–Co(II) complex took about 84 min to complete one oxygenation-deoxygenation cycle. Oxygenation spent about 28 min, whereas deoxygenation took twice times to complete. Kinetics results (Figure 2(a)) showed that the Ala–Co complex could sustain eight continuous oxygenation-deoxygenation cycles over 10 h in an aqueous solution at room temperature. Ser–Co sustained 27 cycles in 20 hours (Figure 2(b)), and His–Co (Figure 2(c)) and Lys–Co (Figure 2(d)) did 550 in 110 h and 20 in 45 h, respectively. Other *α*-amino acid–Co complexes were also examined in the same procedures, and the results are listed in Table 3.

### 3.6. Comparative Study

All 15 *α*-amino acid–cobalt complexes, except for Cys, displayed reversible oxygenation properties but exhibited different affinities to dioxygen; therefore, a systematic comparative study was conducted to reveal the relationship between the structures of amino acids and oxygenation properties of complexes. Ala–Co complex only has a methyl connected with *α*-amino acid group; thus it was used as a reference of other *α*-amino acid–cobalt complexes. In the comparative study, except for reversibility, the times of oxygenation and deoxygenation and number of reversible cycles were involved as the contrast parameters. Some rules were concluded from the comparative study.

## 4. Discussion

All *α*-amino acid–cobalt complexes exhibit reversible binding ability to dioxygen, except for Cys–Co(II). Generally, the oxygenation time (*t*_o_) of a complex is shorter than the deoxygenation time (*t*_d_) for almost all complexes. His–Co yields the minimum *t*_o_ and *t*_d_ of 1 and 7.5 min, respectively. Glu–Co and Asp–Co reach the maximum *t*_o_ and *t*_d_ of 93 and 100 min, respectively. The ligands of the Co complexes were arranged from the shortest to the longest on the basis of the duration dioxygen uptake saturation (*t*_o_: min): His (1), Ser (17), Thr (18), Gly (22), Ala (28), (Pro = Arg) (33), Val (35), Met (37), Lys (42), Asn (50), Asp (83), Gln (87), and Glu (93). Likewise, the ligands of the Co complexes were arranged from the shortest to the longest on the basis of the duration of the complete dioxygen release (*t*_d_: min): His (7.5), Ser (17), Thr (25), Pro (33), Val (50), Ala (56), Met (57), (Arg = Lys) (58), Asn (67), Gly (75), Glu (87), Gln (92), and Asp (100). The His–Co complex requires much less time than the other complexes do in the oxygenation-deoxygenation process; this finding suggested that the His–Co complex is an excellent model of oxygen carriers. According to the theoretical calculation results, *t*_d_ is usually longer than *t*_o_ of these complexes when an H bond forms between ligands and when O_2_ binding occurs.

Another important characteristic parameter to evaluate the oxygenation property of a complex is the number of oxygenation-deoxygenation cycles. Our results suggest that His–Co has the maximum cycle number of 550, whereas Gly displays only 2 cycles. The ligands of the Co complexes were arranged from the highest to the lowest depending on whether they could sustain 5% to 100% of the original oxygenation capacity: His (550), Pro (40), Arg (33), Ser (27), (Glu = Gln) (24), (Val = Lys) (20), Thr (17), Met (16), Asp (12), Asn (11), Ala (8), and Gly (2).

Tables 2 and 3 reveal the results of the comparative analyses of one cycle time (*t*_*T*_, *t*_*T*_ = *t*_o_ + *t*_d_) and cycle numbers (*C*) of 14 *α*-amino acid–cobalt complexes.

Ala–Co complex took 84 min to complete one oxygenation-deoxygenation cycle. This complex could also sustain eight reversible cycles (Table 3). Furthermore, Gly–Co, Val–Co, and Pro–Co (97, 85, and 66 min, resp.) showed a similar cycle time to that of Ala–Co. All of these amino acids have a similar alkyl radical to Ala; thus all these complexes have a similar coordinating structure. Nevertheless, the cycle numbers are decreased in the order of the decrease of numbers of carbon atoms in alkyl chain.

The oxygenation properties of cobalt complexes of His, Ser, and Thr are improved evidently when compared with Ala–Co; these complexes have cycle times of 8.5, 34, and 43 min, as well as reversible cycle numbers of 550, 27, and 17, respectively. In this study, we suppose that this improvement is because they have a heteroatomic group adjacent to their amino acid group. The presence of one more atom from the heteroatomic group (NH_2_ or OH) that coordinates with amino acid, together with Co(II), is helpful to form the complexes and enhance the oxygenation ability.

The Cys–Co complex could bind to dioxygen but shows no reversibility, although it has also one more coordinating atom; this observation is probably because S atom is larger and more basic compared with N and O atoms, which increased the electron density between the metal ions and molecular oxygen, thereby increasing the bond strength of Co–O_2_ and making it more difficult to release dioxygen. This result is consistent with the report that the coordination ability will be modestly increased for metal complexes when a ligand contains S group [38].

In contrast to the Cys–Co complex, the Met–Co complex showed reversible oxygenation patterns similar to that of Ala–Co, with nearly the same time for one oxygenation-deoxygenation cycle (95 min and 16 reversible cycles). This finding may be caused by the fact that the aliphatic S atom cannot coordinate with Co(II) because it is far from the amino acid group; instead, only the *α*-amino acid unit of Met can coordinate with Co(II), and it behaves the same as Ala does. However, the aliphatic S plays a role in the resistance of the complex to autoxidation by increasing its number of reversible cycles to 16.

Arg and Lys have another –NH_2_ group that could act as a potential coordinating group for the ligands. However, –NH_2_ is far from the *α*-amino acid unit as in Met–Co; hence the coordination between the amino acid and Co(II) is much weaker. Thus, the dioxygen affinity of the Co(II) complexes for Arg and Lys is similar to that of Ala–Co, and their cycle times are also almost the same as Ala–Co. The resistance to autoxidation of Arg–Co and Lys–Co is improved by their –NH_2_ group, and the numbers of their reversible cycles increased to 33 and 20, respectively.

Glu and Gln have additional –CONH_2_ and –COOH groups in their structures, respectively, and they exhibit similar oxygenation abilities. The second carbonyl in Glu and Gln can be used as coordination group; however, it does not coordinate with its amino acid group, together with the same Co(II) ion. Instead, this group coordinates with another Co(II) ion to form linear macromolecule during the formation of the Co(II) complexes. Therefore, the times for one oxygenation-deoxygenation cycle of Glu and Gln are extended to 180 and 179 min, respectively, and the numbers of their reversible cycles are also improved to 24.

Asn also contains one more –CONH_2_ group, but it possesses one –CH_2_ group less than Glu in its chain. The carbonyl groups in Asn can promote its coordination with the Co(II) ion. Hence, one cycle time of Asn is 117 min, which is faster than that of Glu. Asp also behaves as Gln, and its one cycle time remains at 183 min, which is nearly the same as that of Gln. Nevertheless, the reversible cycle numbers of both Asp and Asn are retained at 11 and 12, respectively.

All of above 14 oxygenated *α*-amino acid–cobalt complexes have UV-vis absorption. The characteristic absorption peaks of all aliphatic *α*-amino acid–cobalt complexes are similar to one another and appeared at about 365 nm, thereby revealing that oxygenated species of these *α*-amino acid–cobalt complexes have considerable similarity in terms of their coordinating structures and patterns.

Some differences exist between the oxygenated complexes of His–Co and Pro–Co and other oxygenated complexes, and the UV-vis absorption peaks appeared at 374 and 380 nm, respectively. The result is attributed to the fact that both of these amino acids have an aromatic ring that can stabilize the complexes and make the absorption band shift to red waves. Based on these results, the absorption peak at 365 nm would be a characteristic absorption peak for the oxygenated species of the complex. In such case, the UV-vis spectra at 365 nm could be used to characterize the aliphatic oxygenated complexes.

On the basis of the comparative studies, we proposed that the *α*-amino acid group is the basic unit responsible for the reversible oxygenation properties in these 14 complexes. Other functional groups can also affect the rate, cycle times, and other oxygenation properties. The rates and cycle times of the reversible oxygenation process are mainly determined by the coordination ability of amino acids. Groups, such as imidazole in histidine that can cooperate with the *α*-amino acid to form more stable complexes with Co(II), will cause the His–Co complex to exhibit faster oxygenation and deoxygenation rates.

The presence of additional coordinating groups in the amino acids may also affect the oxygenation abilities of the complexes. The presence of –NH_2_ (or –NH) and –OH at a position adjacent to the *α*-amino acid unit could increase both oxygenation-deoxygenation rates and number of reversible cycles. The heteroatom group linked with the chain of the *α*-amino acid can inhibit oxidation and increase the number of cycles.

## 5. Reversible Oxygenation Mechanism

The DFT calculations were conducted for the structural models of the studied compounds, and the results of Ala–Co and His–Co have been reported [28, 39, 40]. Based on the theoretical calculation and experimental results, the oxygenation mechanism of the Co(II) complexes is proposed as follows.

When a complex binds to dioxygen, the* d*-orbitals of Co(II) are split, and the distribution of the electrons on the 3*d* orbital is t_2g_^6^e_g_^1^. For the oxygenated complexes, the energy level of e_g_ orbitals of Co(II) is fairly close to the energy level of *π* ^*∗*^ orbitals for dioxygen. Therefore, the electron of e_g_ orbitals can transfer to *π* ^*∗*^ orbitals of dioxygen to form the Co–O_2_ bond [24].

**Figure 3:**
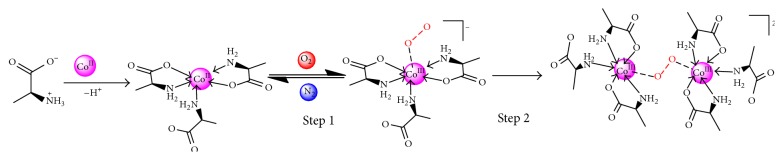
Formation and oxygenation of the Ala–Co complex.

**Figure 4:**
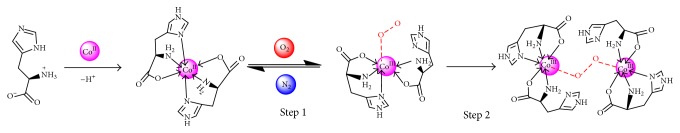
Formation and oxygenation of the His–Co complex.

The *α*-amino acid–Co(II) complexes can reversibly bind to O_2_ depending on the co-coordination of *α*-amino and carboxyl groups. The electronegativity of N atom is smaller than that of O atom, and its lone pair electrons in N are closer to the central Co(II); as a result, the electron cloud density on Co(II) is increased. This phenomenon is helpful to transfer the e_g_^1^ electron from Co(II) to the *π* ^*∗*^ orbit of O_2_ and form the Co–O_2_ bond when O_2_ coordinates with the Co(II) complex. According to the theoretical calculation, before and after oxygenation, the bond lengths of N–C, C–O, Co–O, and Co–N in the complexes shortened; for Ala–Co [39], Co–O and Co–N have 2.0889–2.266 and 2.2131–2.2137 lengths in the complex, respectively; after oxygenation, Co–O and Co–N become 1.941–1.984 and 2.1368–2.2059, respectively. For His–Co [40], Co–O, Co–N, and Co–N (imidazole) are 2.2723, 2.0113 and 2.0775, 2.2847, and 1.9847, 1.9910, respectively, in the complex; after oxygenation, Co–O, Co–N, and Co–N (imidazole) become 1.9519, 1.9286, 1.9361, 2.0006, and 1.9632, 1.9580, respectively. These results showed that the *α*-amino and carboxyl groups have conjugation in oxygenation. The conjugation of coordinated carboxyl can make Co–O_2_ bond more stable where the peroxo complex forms. With the transition of the electron and conjugation, the O_2_ binding becomes reversible when O_2_ and N_2_ atmospheres are alternatively changed. The reversible oxygenation of the complexes would occur, as shown in Figures 3 and 4.

## 6. Conclusions

Our study revealed that the structural detail of *α*-amino acid plays a key role in determining the reversible oxygenation/deoxygenation ability of the complexes formed by Co(II) and amino acid. We observed that the auxiliary groups linked to the *α*-amino acid group can affect the affinities of the complexes to dioxygen and their abilities to undergo antiautoxidation. In particular, the presence of –NH_2_ (or –NH) or –OH group at a position adjacent to the amino acid unit enhances the oxygenation-deoxygenation rates and number of reversible cycles. A heteroatom group linked to the chain of the amino acid improves the resistance to oxidation and may increase the number of reversible cycles. Therefore, a reversible oxygenation mechanism of amino acid–Co(II) complexes is proposed, that is, the coaction of the strong electron donor of the amino group, and conjugation of the carboxyl group is an important phenomenon of the reversible oxygenation of these complexes. This strategy may provide a useful basis of novel oxygen carriers.

## Supplementary Material

Supplementary Material containing supporting data of oxygenation properties of natural *α*-amino acid-Co complexes.

## Figures and Tables

**Table 1:** Concentrations of 15 amino acids and cobalt salts.

L^①^	Gly	Ala	Val	Ser	Thr	His	Pro	Met	Cys	Arg	Lys	Glu	Gln	Asn	Asp
Mr	75	89	117	105	119	155	115	149	121	174	146	147	146	132	133
*c* ^*②*^_Co_ × 10^−3^	6.0	0.33	0.5	1.0	1.0	0.028	0.33	1.0	0.1	0.25	0.2	1.0	1.0	2.0	2.0
*c*_L_ × 10^−3^	18.0	1.0	1.5	3.0	3.0	0.056	1.0	3.0	0.3	0.75	0.6	3.0	3.0	6.0	6.0

Notes: ① amino acids ligands; ②  *c*: mol·L^−1^.

**Table 2:** Suitable pH, *λ*_max⁡_, and reversibility for the dioxygen uptake of 15 complexes.

L	Gly	Ala	Val	Ser	Thr	His	Pro	Met	Cys	Arg	Lys	Glu	Gln	Asn	Asp
*λ*_max⁡_	362	365	368	368	366	320/374	380	365	347/442	366	365	365	365	365	365
pH^③^	8.5	9.5	9.0	9.5	9.5	8.0	10.5	10.0	7.0	9.5	10.5	10.5	10.0	10.0	11.5
OU^④^	Yes	Yes	Yes	Yes	Yes	Yes	Yes	Yes	Yes	Yes	Yes	Yes	Yes	Yes	Yes
UV^⑤^	Yes	Yes	Yes	Yes	Yes	Yes	Yes	Yes	No	Yes	Yes	Yes	Yes	Yes	Yes
OX^⑥^	Yes	Yes	Yes	Yes	Yes	Yes	Yes	Yes	No	Yes	Yes	Yes	Yes	Yes	Yes

Notes: ③ suitable pH value for the test; ④ oxygen uptake performance; ⑤ reversible performance tested by UV-vis spectrum; ⑥ reversible performance tested by oxygen electrode.

**Table 3:** Oxygenation parameters of 15 amino acid complexes.

L	Gly	Ala	Val	Ser	Thr	Pro	His	Cys	Met	Arg	Lys	Glu	Gln	Asn	Asp
*t*_o_^⑦^	22	28	35	17	18	33	1	2	37	33	42	93	87	50	83
*t*_d_^⑧^	75	56	50	17	25	33	7.5	/	57	58	58	87	92	67	100
*t*_*T*_^⑨^	97	84	85	34	43	66	8.5	/	95	91	100	180	179	117	183
*C* ^*⑩*^	2	8	20	27	17	40	550	/	16	33	20	24	24	11	12

Notes: ⑦ *t*_o_ for oxygenation time (minutes); ⑧ *t*_d_ for deoxygenation time (minutes); ⑨ *t*_*T*_ for one oxygenation-deoxygenation circulation; units: minutes; ⑩  *C*: cycle numbers.
