# Supplementary material for: Reversible Oxygenation of α-Amino Acid–Cobalt(II) Complexes
Source: Bioinorg Chem Appl. 2016 Feb 28;2016:3585781. doi: 10.1155/2016/3585781 (PMC4789021; doi:10.1155/2016/3585781)
Supplement: Supplementary file 1 — Supplementary Material containing supporting data of oxygenation properties of natural α-amino acid-Co complexes. [file 3585781.f1.doc]

# REVERSIBLE OXYGENATION OF *α*-AMINO ACID–COBALT(II) COMPLEXES

**Xincun Zhang**^1^**, Fan Yue**^1^**, Hui Li**^1^**, Yan Huang**^1^**,Yi Zhang**^2^**, Hongmei Wen**^1^**, Jide Wang**^1^*****

^1^ Key Laboratory of Oil and Gas Fine Chemicals, Ministry of Education and Xinjiang Uyghur Autonomous Region, College of Chemistry and Chemical Engineering, Xinjiang University, Urumqi 830046, Xinjiang, China

^2^ College of Chemistry and Chemical Engineering, Central South University, Changsha 410083, Hunan, China


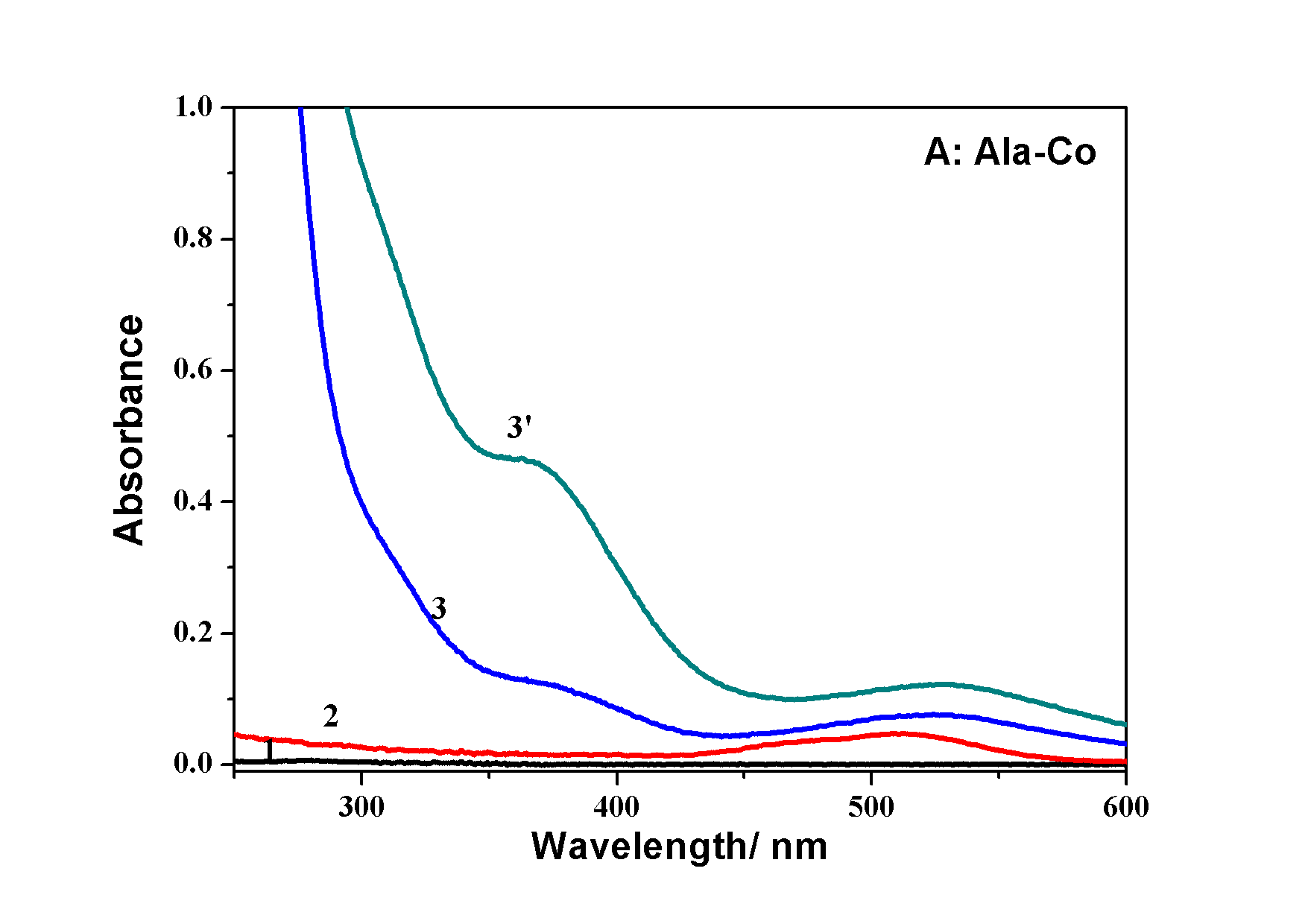

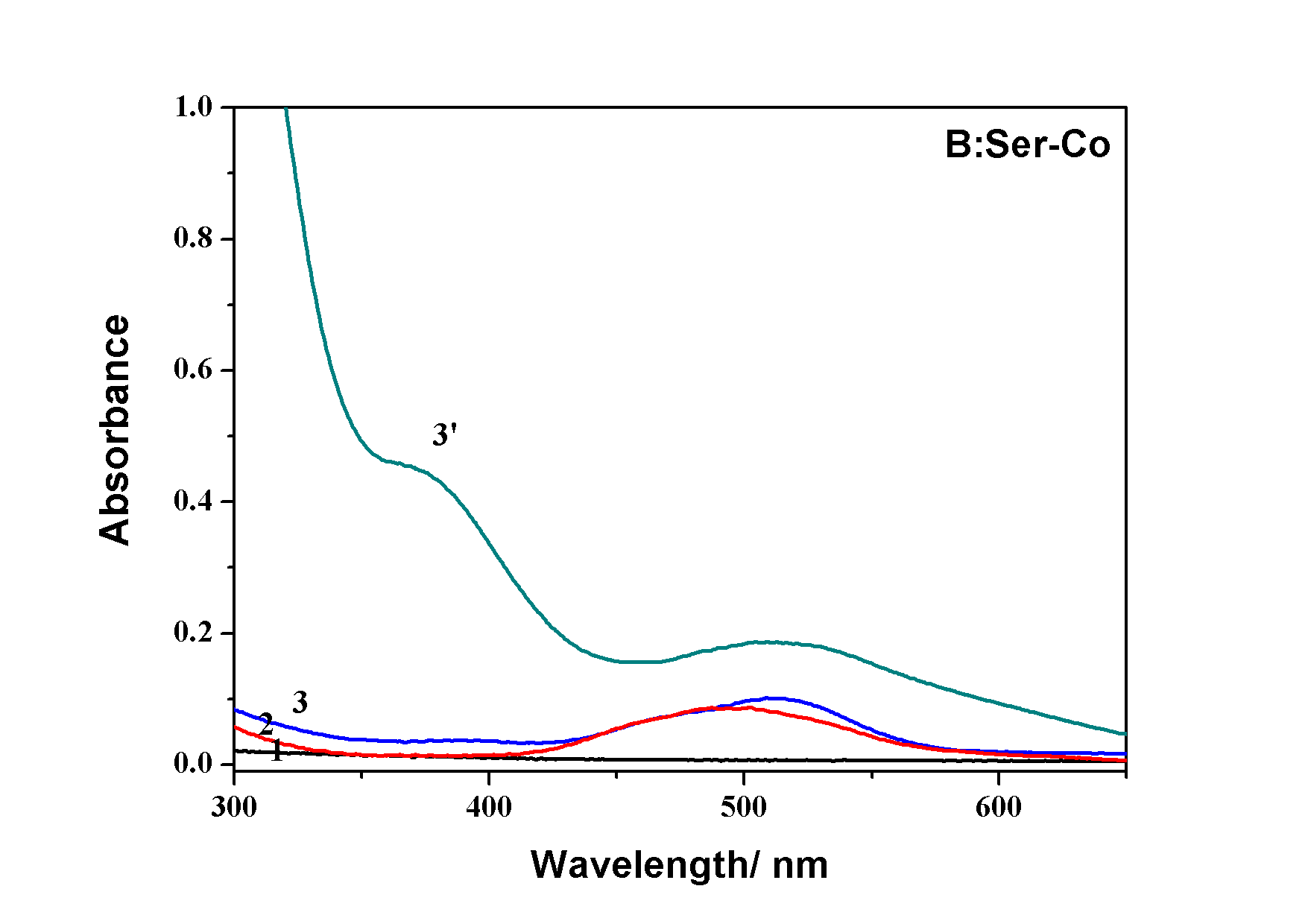

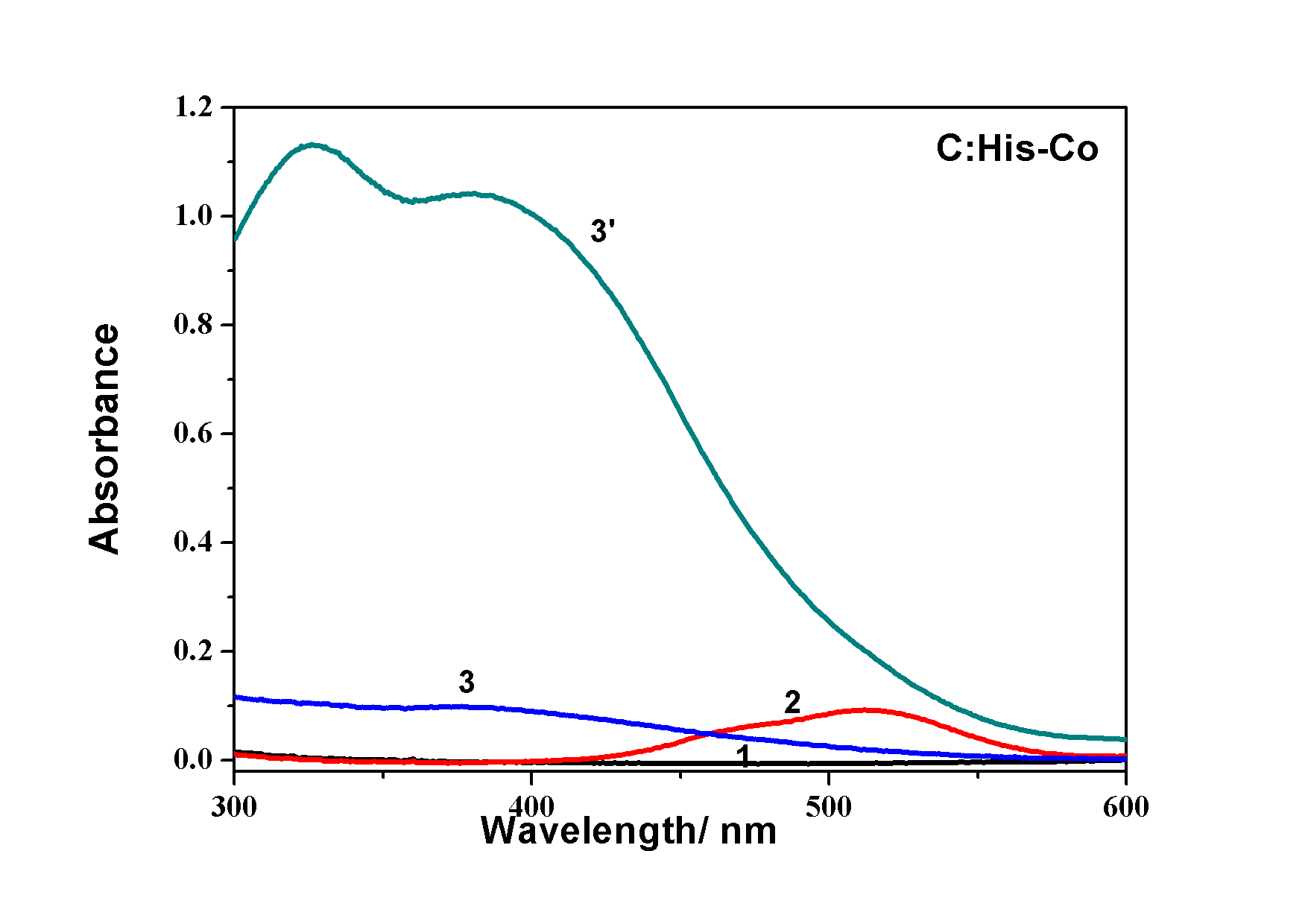

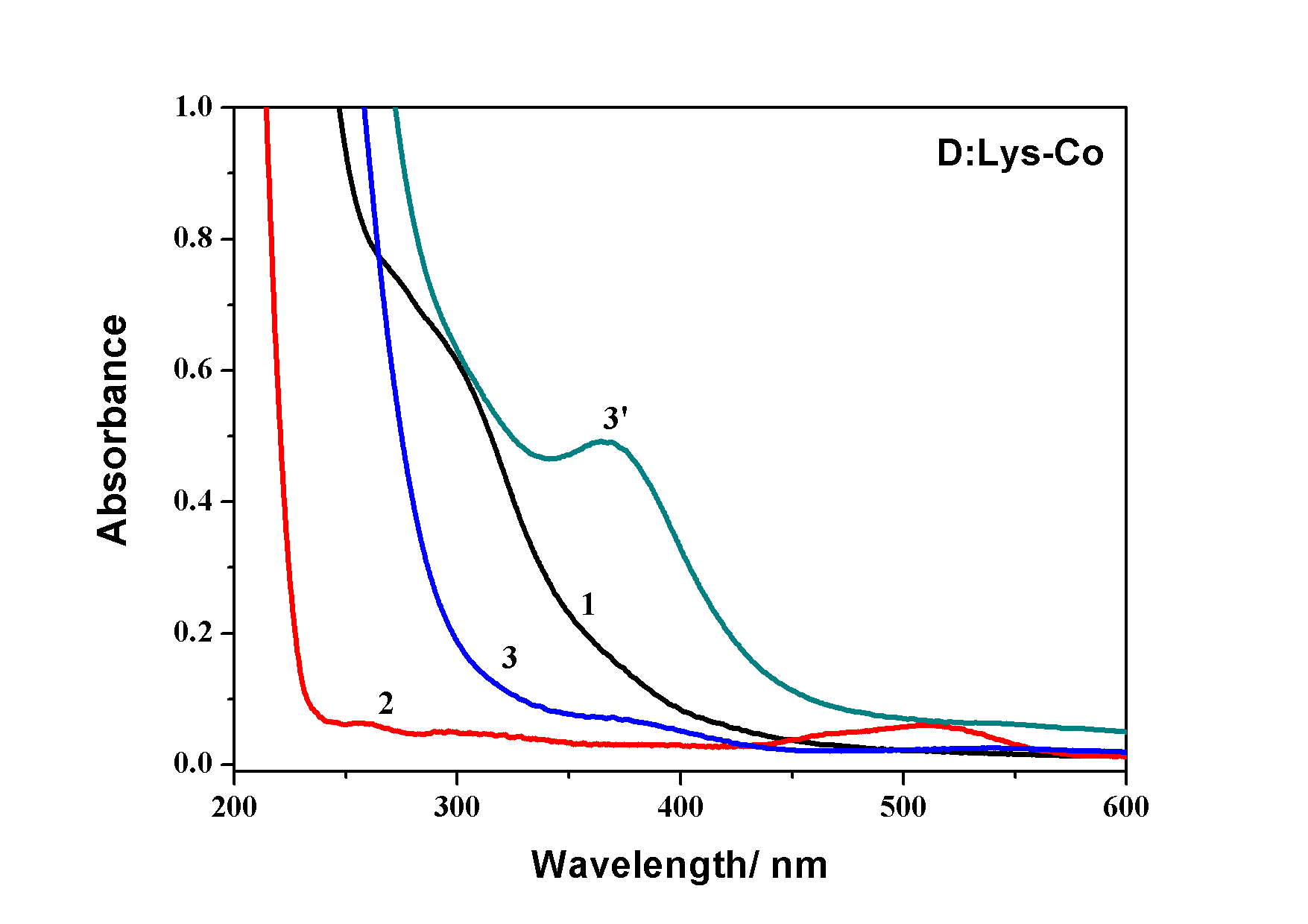


Fig. S1 The UV-Vis spectral of amino acids, Co(II), complexes in N_2_ atmosphere and generated oxygenated complexes in aqueous solution at room temperature. A: Ala-Co; B: Ser-Co; C: His-Co; D: Lys-Co (curve 1: L, 2: Co(II), 3: L-Co complexes in N_2_; 3' Oxygenated complexes)

Fig .S 2 The IR spectra of amino acids and corresponding complexes; Ala-Co , Ser-Co, His-Co and Lys-Co (curve 1: L, curve 2: L-Co)


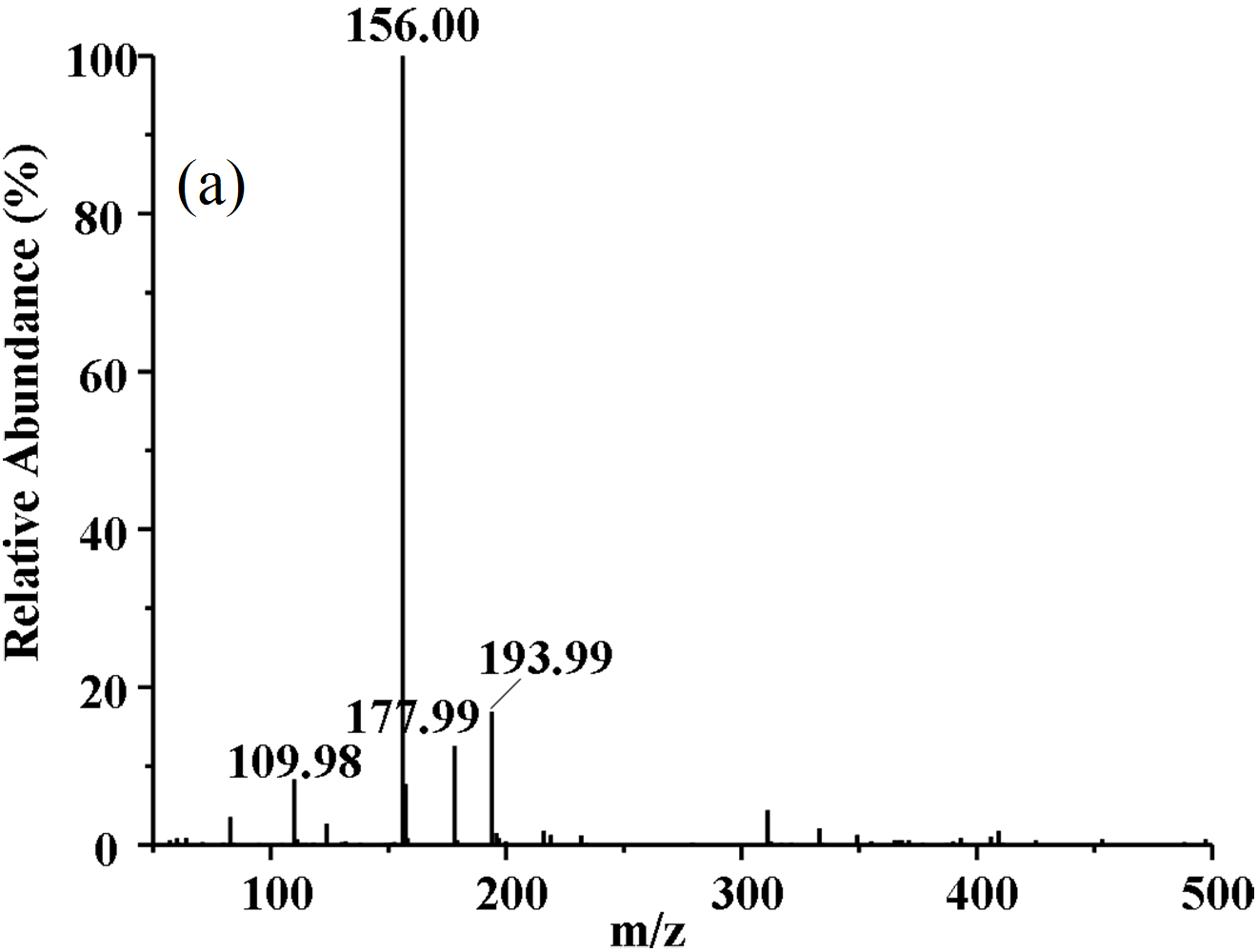

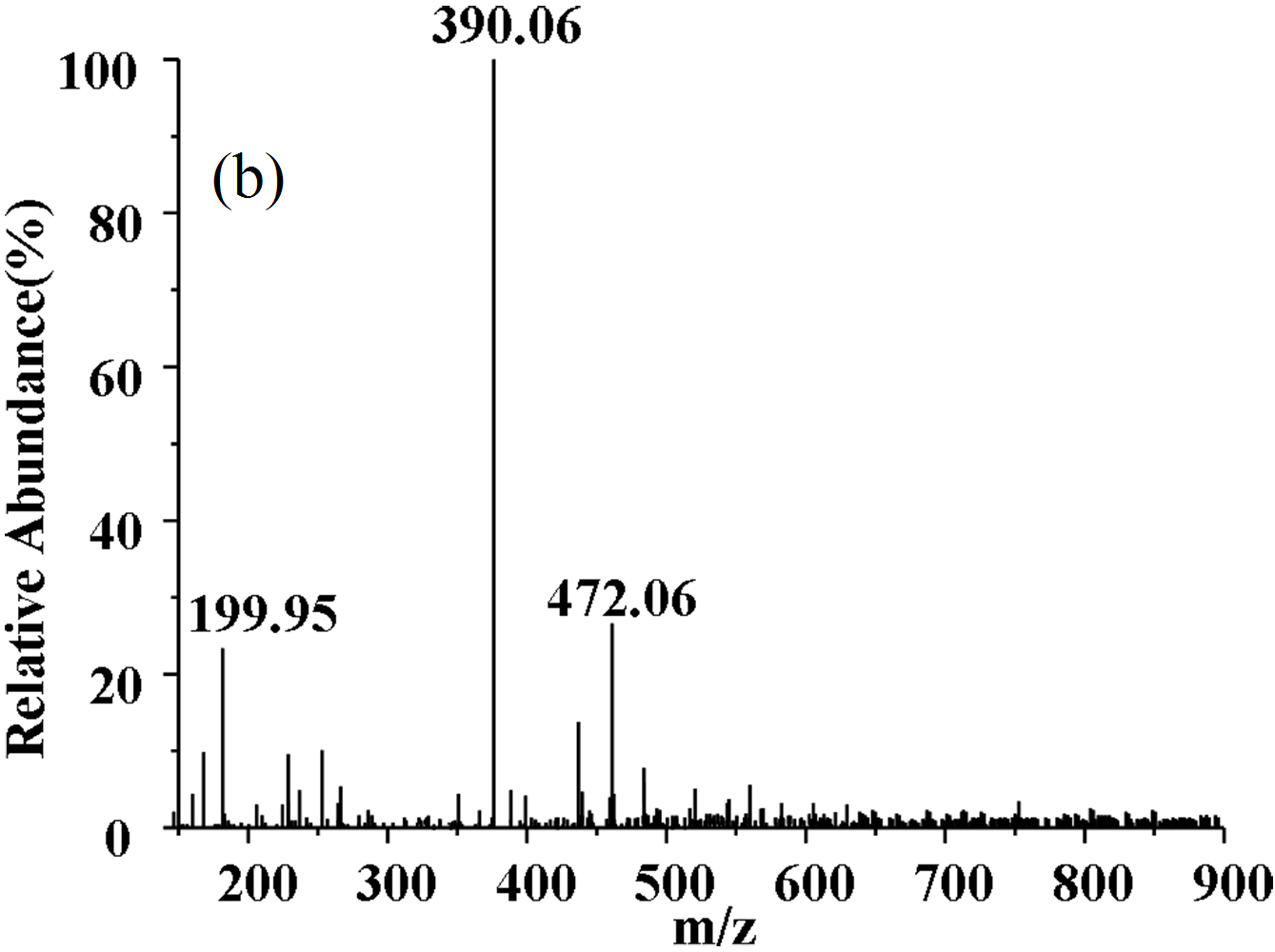


Fig. S3 The positive ESI full scan mass spectra of His (A) and His -cobalt complex(B)


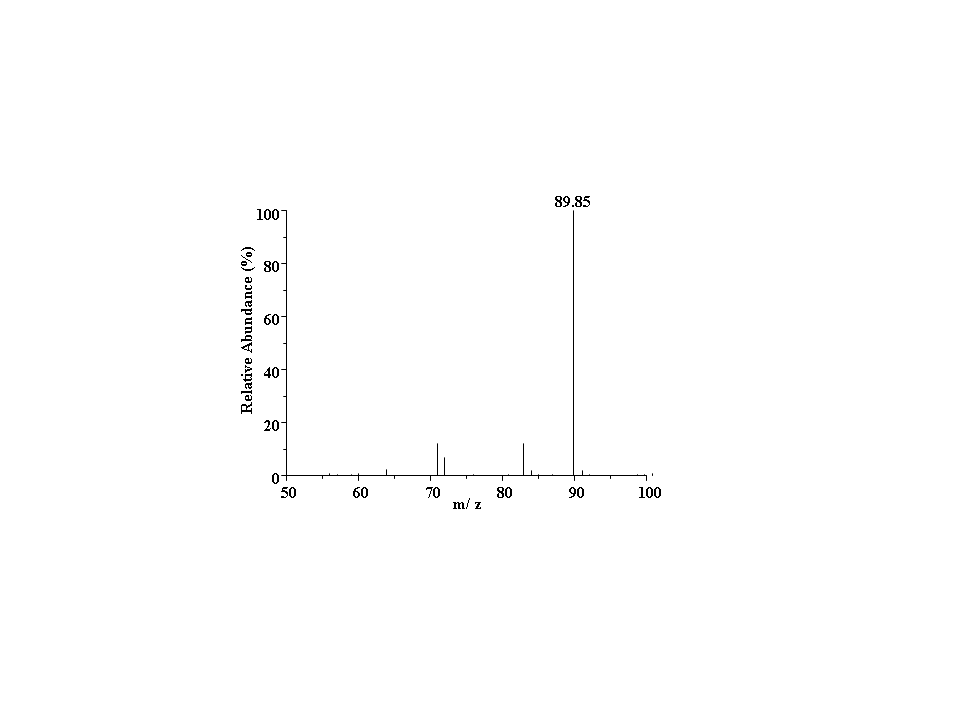

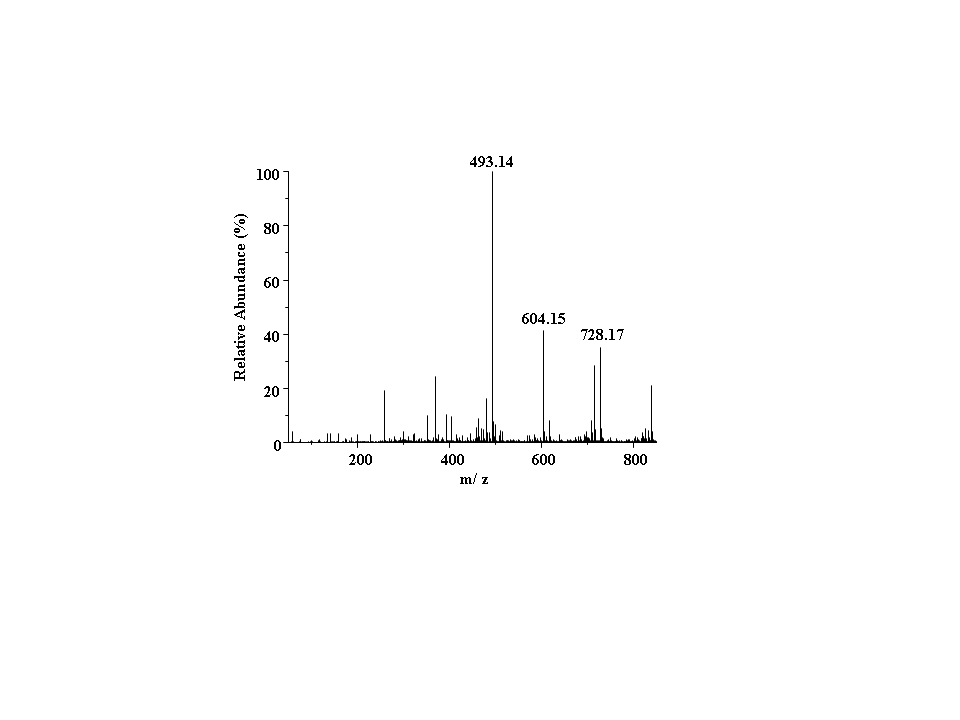


(a)

(b)

Fig.S4 The positive ESI full scan mass spectra of Ala (A) and Ala -cobalt complex (B)

Fig. S5 A-pH curves of L-Co (curve 1, 2, 3 and 4 for Ala, Ser, His and Lys-Co, respectively )


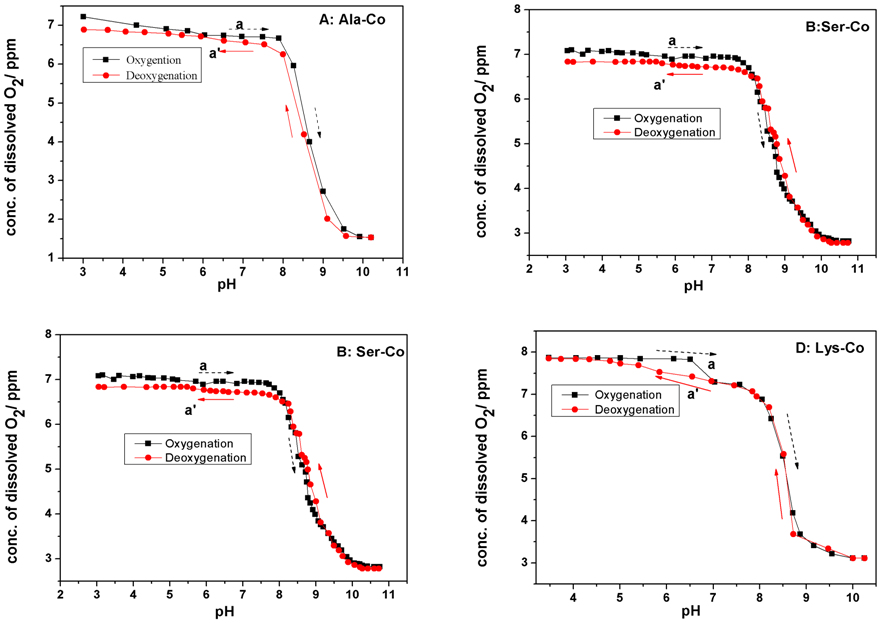


Fig .S6 Relationship between concentration of dissolved O_2_ and pH values (a) from 3 to 11 and (a') from 11 to 3
